# Identification of candidate receptors mediating the osteoanabolic influence of Wnt1

**DOI:** 10.1093/jbmrpl/ziag153

**Published:** 2026-09-09

**Authors:** Kim M Walter, Leon Grahn, Wenbo Zhao, Kenneth Schöneck, Sandra Paradies, Julia Luther, Mubashir Ahmad, Melanie Haffner-Luntzer, Anita Ignatius, Ernesto Bockamp, Michael Amling, Thorsten Schinke, Timur A Yorgan

**Affiliations:** Department of Osteology and Biomechanics, University Medical Center Hamburg-Eppendorf, 20246 Hamburg, Germany; Department of Osteology and Biomechanics, University Medical Center Hamburg-Eppendorf, 20246 Hamburg, Germany; Department of Osteology and Biomechanics, University Medical Center Hamburg-Eppendorf, 20246 Hamburg, Germany; Department of Osteology and Biomechanics, University Medical Center Hamburg-Eppendorf, 20246 Hamburg, Germany; Department of Osteology and Biomechanics, University Medical Center Hamburg-Eppendorf, 20246 Hamburg, Germany; Department of Osteology and Biomechanics, University Medical Center Hamburg-Eppendorf, 20246 Hamburg, Germany; Institute of Orthopedic Research and Biomechanics, University Medical Center Ulm, 89081 Ulm, Germany; Institute of Orthopedic Research and Biomechanics, University Medical Center Ulm, 89081 Ulm, Germany; Institute of Orthopedic Research and Biomechanics, University Medical Center Ulm, 89081 Ulm, Germany; Institute for Translational Immunology and Research Center for Immunotherapy, University Medical Center, Johannes Gutenberg University, 55131 Mainz, Germany; Department of Osteology and Biomechanics, University Medical Center Hamburg-Eppendorf, 20246 Hamburg, Germany; Department of Osteology and Biomechanics, University Medical Center Hamburg-Eppendorf, 20246 Hamburg, Germany; Department of Osteology and Biomechanics, University Medical Center Hamburg-Eppendorf, 20246 Hamburg, Germany

**Keywords:** Wnt-signaling, Osteoblasts, Genetic animal models, Remodeling, Wnt1, Fzd, Bone histomorphometry

## Abstract

Although WNT signaling pathways are of central importance for osteoblast regulation, Wnt1 is the only known ligand for which an osteoanabolic function has been demonstrated based on human genetic evidence. The therapeutic potential of Wnt1 was further documented in a transgenic model allowing inducible *Wnt1* expression in osteoblasts, which produced a marked increase in trabecular and cortical bone mass within three weeks. However, the molecular mechanisms and target cells of Wnt1 action remain unclear. In this study, we analyzed transgenic mice with Wnt1 induction for 3, 5, or 7 d and confirmed rapid osteoanabolic effects in the spine and tibia. We also observed strong recruitment of osteoblasts and progenitor cells to bone surfaces. Cell culture experiments revealed that Wnt1 primarily acts on mesenchymal progenitor cells, as only the mesenchymal cell line ST2, not MC3T3-E1 osteoblasts, responded to Wnt1. Comparing receptor expression between these lines identified several *Fzd* genes, including *Fzd4*, with higher expression in ST2 cells. Knockdown by siRNA confirmed that FZD4, together with LRP5 and LRP6, mediates Wnt1-responsiveness in ST2 cells. Although *Fzd4* deletion did not impair the in vivo anabolic effects of Wnt1 overexpression, *Fzd4*-heterozygous mice displayed moderately reduced bone mass at 24 wk. These findings demonstrate that Wnt1 strongly influences mesenchymal osteoprogenitor cells and identify FZD4 as one candidate receptor mediating these effects.

## Introduction

Since osteoporosis represents one of the most prevalent diseases in the elderly population with a strong impact on the quality of life, the identification of targets for osteoanabolic treatment strategies is of paramount importance. Here, the WNT signaling pathway, with its 19 WNT ligands, 10 receptors from the Frizzled (FZD) family and numerous supporting molecules, can be considered a highly potent and promising molecular regulator of bone metabolism. The combined research efforts of the last decades have resulted in the identification of several WNT pathway components with osteologic relevance. The initial finding linking WNT signaling to skeletal biology was however related to a transmembrane protein known as LRP5, which had an established function as a co-receptor for WNT ligands.^1^ More specifically, whereas activating variants of *LRP5* were identified in patients with osteosclerosis, biallelic loss-of-function variants of *LRP5* were found to cause osteoporosis pseudoglioma syndrome, a severe childhood disorder with co-existence of impaired bone formation and blindness.^1^^,^^2^

The impact of the WNT signaling pathway on skeletal cell types was subsequently analyzed by various methods, including a large number of genetically modified mouse models and evidence from human genetics. These efforts resulted in the identification of several relevant molecules, two of which can be considered particularly important. One of them is Sclerostin, a physiological inhibitor of bone formation acting as an LRP5 antagonist, for which a neutralizing antibody has been developed as an osteoanabolic treatment for osteoporosis.^3–5^ The other molecule is Wnt1, for which biallelic loss-of-function variants were found to cause osteogenesis imperfecta type XV.^6–8^ Although initially considered surprising, since *Wnt1* inactivation in mice had been reported to primarily affect brain development, the osteoanabolic function of Wnt1 was subsequently demonstrated by various studies.^9–15^ This also included genetically modified mouse models with either cell-type specific *Wnt1* inactivation, introduction of pathogenic genetic *Wnt1* variants or inducible *Wnt1* over-expression.

Particularly, the latter mouse model, in which the presence of two transgenes allows to induce *Wnt1* expression specifically in osteoblasts by doxycycline removal from the food, was very informative to demonstrate the osteoanabolic action of Wnt1. More specifically, we observed a remarkable increase of trabecular and cortical bone mass after 3 wk of *Wnt1* induction in this model, as well as a positive influence on skeletal fracture healing.^11^^,^^16^ Interestingly, however, when combining this model of inducible *Wnt1* expression with *Lrp5*-deficient mice, the bone mass increase after doxycycline removal was not reduced, suggesting that other co-receptors, potentially LRP6, are required for the osteoanabolic function of Wnt1.^11^^,^^17^ Unfortunately, however, we and others did not succeed so far to identify the most critical receptor complex responsible for transmitting the Wnt1 signal into increased bone formation. Since FZD receptors, due to their serpentine structure, are principally regarded as suitable drug targets, we consider the search for such a receptor as an important goal of future studies. Furthermore, the cells that directly react to Wnt1 and mediate the strong osteoanabolic response have not been conclusively identified yet.

Here, we show, by a combination of in vivo and cell culture experiments, that Wnt1 primarily acts on osteoblast progenitor cells. Taking advantage of the mesenchymal cell line ST2, we show that the responsiveness to Wnt1 is only blunted by simultaneous siRNA-mediated knockdown of both putative co-receptors, namely *Lrp5* and *Lrp6*. Moreover, since siRNA-mediated knockdown of *Fzd4* partially reduced the response of ST2 cells to Wnt1, and since *Fzd4* was barely expressed in Wnt1-unresponsive MC3T3-E1 cells, we focused on FZD4 as a putative osteoanabolic Wnt1 receptor. Although *Fzd4* deficiency did not significantly impair the bone mass increase following *Wnt1* induction in transgenic mice, we identified a moderate trabecular bone mass reduction in 24-wk-old *Fzd4*-heterozygous animals. Especially, this latter finding is potentially relevant, since heterozygous pathogenic variants of *FZD4* lead to the same ocular pathologies as described for individuals with heterozygous *LRP5* inactivation.^18^^,^^19^

## Materials and methods

### Animal generation and husbandry

In this study, mice with an inducible, osteoblast-specific transgene were utilized (STOCK-Tg(tetO-*Wnt1*,-luc)TWNTLach-Tg(Col1a1-tTa)139Niss/Uke. This mouse line was previously established at our institution.^11^ FZD4-deficient mice (B6;129-*Fzd4*^tm2.1dNat^/J) were obtained from the Jackson Laboratory (strain number 011078).^20^ The *Wnt1*-transgene was introduced into the *Fzd4*-deficient mice by cross-breeding in order to establish the line STOCK-Tg(tetO-*Wnt1*,-luc)TWNTLach-Tg(Col1a1-tTa)139Niss-*Fzd4*^tm2.1dNat^/Uke. Unless indicated otherwise, mice from lines harboring the *Wnt1*-transgene were kept under a doxycycline-containing diet (ssniff-Spezialdiäten GmbH). The expression of the transgene was induced by changing the diet to regular rodent chow (1328P, Altromin Spezialfutter GmbH & Co. KG). Genotyping was performed by PCR using the primer pair 5′-CGC CAA AAA CAT AAA GAA AGG C-3′ and 5′-TGT CCC TAT CGA AGG ACT CTG G-3′ for Wnt1-Tg, 5′-CTC TGC ACC TTG GTG ATC-3′ and 5’-GCT GCT TAA TGA GGT CGG-3′ for ColtTA-Tg, 5′-CAG CTG ACA ACT TTC ACG CC-3′ and 5′-CTG GAA GGG CGA AGT TCT GT-3′ for the *Fzd4* WT, and 5′-TGT AAA CCC CAA ATA CCG TG-3′ and 5′-CGT ATA ATG TAT GCT ATA CGA AG-3′ for the *Fzd4* deficiency allele. As this was an explorative study, the sample size was not statistically determined a priori but based on prior experience and animal availability. Beyond genotype and sex, no additional criteria were applied for including or excluding animals in this study. No animals were excluded from the analysis. No adverse events were observed. Cages for different *Wnt1*-transgene induction times were chosen randomly. Investigators were blinded during outcome assessment.

All mice were housed in the same specific pathogen-free environment with a 12-h light/dark cycle, 45%-65% relative humidity and 20-24 °C ambient temperature in open or individually ventilated cages with wood shavings bedding and nesting material in groups not surpassing 6 animals. Mice had ad libitum access to tap water and chow. After induction of the transgene, the welfare of mice was assessed daily based on overall appearance and body weight. Prior to sacrifice by CO_2_ intoxication, animals were anesthetized by exposure to 80% CO_2_/20% O_2_. The success of the procedure was ensured by cardiac exsanguination. Torso length was determined after fixation.

### Histology

For histology, skeletons were fixed in 3.7% PBS-buffered formaldehyde for 48 h before they were transferred into 80% ethanol. The lumbar vertebral bodies L1-L4 and the left tibia of each mouse were dehydrated in ascending alcohol concentrations, before they were embedded into methylmethacrylate. Sections of 4 μm thickness were cut in the sagittal plane using a Microtec rotation microtome (Techno-Med GmbH). Sections were stained by von Kossa/van Gieson and toluidine blue staining procedures as described previously.^21^ Histomorphometry was carried out according to the guidelines of the American Society for Bone and Mineral Research^22^ using Bioquant Osteo (BIOQUANT Image Analysis Corp.) and OsteoMeasure (Osteometrics Inc.) systems. Overview images were acquired through the Axiovision software with an Axiocam mounted to an Axioscope microscope equipped with an EC Plan Neofluar 1.25× objective lens (numerical aperture 0.03) at room temperature with air as the imaging medium (all components: Carl Zeiss AG). Higher magnification images were acquired with an EVOS M5000 integrated imaging system (Thermo Fisher Scientific Inc.) equipped with an LCPlanFl 20x, numerical aperture 0.40, objective lens (Olympus K.K.) at room temperature with air as the imaging medium.

### Micro-CT (μCT) analysis

For μCT analysis, the right tibia was fixed and placed into a radiotranslucent sample holder. Scanning was performed as previously described^23^ with a voxel resolution of 10 μm at 55 kVp, 145 μA using a μCT 40 desktop cone-beam micro-CT (Scanco Medical) according to standard guidelines.^24^ The Scanco MicroCT software suite (v6.5-2, Scanco Medical AG) was used for evaluation. Femoral cortical bone was analyzed in a 1000 μm long volume situated in the middle of the diaphysis. Cortical bone evaluation was performed with a threshold of 300. Trabecular bone was analyzed in the distal metaphysis of the femur in a volume situated 2500-500 μm proximal of the distal growth plate with a threshold of 250. For visualization of the proximal tibial metaphysis, simple 3D segmentation based on a threshold of 300 was applied on a volume situated 6000-2000 μm distal of the proximal growth plate.

### Cell culture

The mesenchymal cell line ST2 (ACC 333) and the pre-osteoblastic cell line MC3T3-E1 (ACC 210) (both obtained from DSMZ) were cultured in RPMI 1640 (Thermo Fisher Scientific) or α-MEM (Merck KGaA), respectively. The cell lines were not further authenticated after delivery and mycoplasma tests remained negative throughout the project. Primary calvarial osteoblasts were isolated by sequential collagenase-dispase II digestion from the calvariae of 5-day-old C57Bl/6J mice and cultured in α-MEM (Merck KGaA). All cell culture media were supplemented with 10% FCS (Thermo Fisher Scientific) and 100 U/mL penicillin-streptomycin (Thermo Fisher Scientific). For osteogenic differentiation, 50 μg/mL ascorbic acid and 10 mM β-glycerophosphate (both Sigma-Aldrich Corp.) were added to the medium 2 d after plating. The medium was changed every 2-3 d. To investigate the effect of Wnt1, commercially available murine recombinant Wnt1/Sfrp1 complex (100 μg/mL) (Bio-Techne Corp.) or the corresponding volume of carrier (PBS) were added to α-MEM (Merck KGaA). Alizarin red staining was performed after 10 or 15 d of differentiation as previously described.^25^ For the evaluation of immediate effects on gene expression, cells were serum starved overnight and stimulated with 100 ng/mL recombinant Wnt1/Sfrp1 complex (9765-WN-010, Bio-Techne Corp.), 50 ng/mL recombinant Wnt3a (315-20-10UG, Thermo Fisher Scientific Inc.) or PBS for 6 h. Gene knockdown in cell cultures was performed by RNA interference utilizing the following commercially available products: Lrp5 (sc-149050, Santa Cruz Biotechnology, Inc.); Lrp6 (sc-37234, Santa Cruz Biotechnology, Inc.); Fzd4 (sc-39984, Santa Cruz Biotechnology, Inc.); Fzd6 (EMU044771, Merck KGaA, Germany); Fzd10 (sc-39997, Santa Cruz Biotechnology, Inc.); and control siRNA (sc-37007, Santa Cruz Biotechnology, Inc.). Cells were seeded in 24-well format at 30.000 cells/well and transfected with 5 pmol of siRNA utilizing Lipofectamine RNAiMAX (Thermo Fisher Scientific Inc.) reverse transfection according to the manufacturer’s instructions. Stimulation with 100 ng/mL recombinant Wnt1/Sfrp1 complex (Bio-Techne Corp.) or PBS for 6 h was initiated 48 h after transfection.

### Expression analysis

RNA isolation was performed using the Nucleospin RNA II kit (Macherey-Nagel GmbH & Co. KG). The concentration of RNA was measured using a NanoDrop ND-1000 system (Thermo Fisher Scientific Inc.). RNA quality and integrity was assessed by the TapeStation 2200 system (Agilent Technologies Inc.). For qRT-PCR expression analysis, the RNA was reverse transcribed using the Verso cDNA Synthesis Kit (Thermo Fisher Scientific Inc.) according to manufacturer instructions. The quantitative expression analysis was performed using the StepOnePlus system and predesigned TaqMan gene expression assays (Thermo Fisher Scientific Inc.): *Ahr* (Mm00478932_m1), *Apcdd1* (Mm01257559_m1), *Axin2* (Mm00443610_m1), *Enpp2* (Mm00516572_m1), *Fzd1* (Mm00445405_s1), *Fzd2* (Mm02524776_s1), *Fzd3* (Mm00445423_m1), *Fzd4* (Mm00433382_m1), *Fzd5* (Mm00445623_s1), *Fzd6* (Mm00433387_m1), *Fzd7* (Mm00433409_s1), *Fzd8* (Mm01234717_s1), *Fzd9* (Mm01206511_s1), *Fzd10* (Mm00558396_s1), *Lrp5* (Mm01227476_m1), *Lrp6* (Mm00999795_m1), and *Nkd2* (Mm00472240_m1). *Gapdh* (4352661) expression was used as an internal control. Relative quantification was performed according to the ΔΔCT method for fold change data or related directly to Gapdh (2^−CT(target)^/2^−CT(Gapdh)^). Whole transcriptome analysis was performed by microarray with RNA from 3 independent experiments per condition utilizing the Clariom S, mouse system (Thermo Fisher Scientific Inc.) according to the manufacturer’s GeneChip WT PLUS reagent kit manual (document 703174, revision A.0). In brief, 100 ng of total RNA from per sample were used for the synthesis of second cycle ss-cDNA. 5.5 μg of fragmented and labeled cDNA was subsequently used for gene chip hybridization. After washing and staining with the Affymetrix Fluidics Station 450. Microarrays were scanned with the Affymetrix Gene Chip Scanner 7G, and the signals were analyzed with the Transcriptome Analysis Console software (TAC 4.0.3, Thermo Fisher Scientific Inc.) using default analysis settings (version 2) and Gene + Exon − SST-RMA as summarization. The full datasets are available at the Gene Expression Omnibus repository (accession number GSE311746).^26^

### Immunological assays

For Western blotting, ST2 and MC3T3-E1 cells were harvested and lysed in PBS (pH 7.4) containing 0.1% Triton X-100 and protease inhibitors for 1 h at 4 °C. Aliquots of cell extracts (20 μg protein) were separated by SDS-PAGE under reducing conditions and analyzed by Western blotting with specific antibodies against FZD4 (R&D Systems Inc., MAB194, 1:500) and Gapdh (Proteintech, 60004-1-Ig, 1:1000), the latter serving as loading control. After incubation with secondary HRP-conjugated goat anti-rat (FZD4) and goat anti-mouse (Gapdh), the immunoreactive bands were visualized by enhanced chemiluminescence. Since the Gapdh signals differed between both cell lines, Ponceau S staining Thermo Fisher Scientific Inc., A40000279) was used as an additional loading control.

Serum concentrations of murine osteocalcin were determined with a commercially available enzyme linked immunosorbent assay (Biomedical Technologies Inc., BT-470) according to the manufacturer’s instructions.

### Statistics

Data were analyzed by 2-tailed Student’s *t-*test using Excel software (Microsoft Corp.) when 2 conditions were compared. For four groups, a one-way ANOVA with Tukey’s multiple comparison test was applied (GraphPad Software Inc.). When comparing four groups with different alleles of 2 independent genetic loci, a 2-way ANOVA with Sidak’s multiple comparison test was used (GraphPad Software Inc.). Observed vs expected frequencies were compared by χ^2^ test using Prism software (GraphPad Software Inc.). All data are reported as mean ± SD with additional points representing individual animals. A value of *p* ≤ .05 was considered as statistically significant. All data have been provided within the manuscript.

### Study approval

All animal experiments were performed in accordance with the local implementation of EU Directive 2010/63/EU for animal experiments approved by the Ethic Committee of the University Medical Center Hamburg-Eppendorf and by the “Behörde für Justiz und Verbraucherschutz” (N022/2022, N059/2022).

## Results

### Short-term *Wnt1* induction in osteoblasts triggers recruitment of osteoblasts to existing bone surfaces

**Figure 1:**
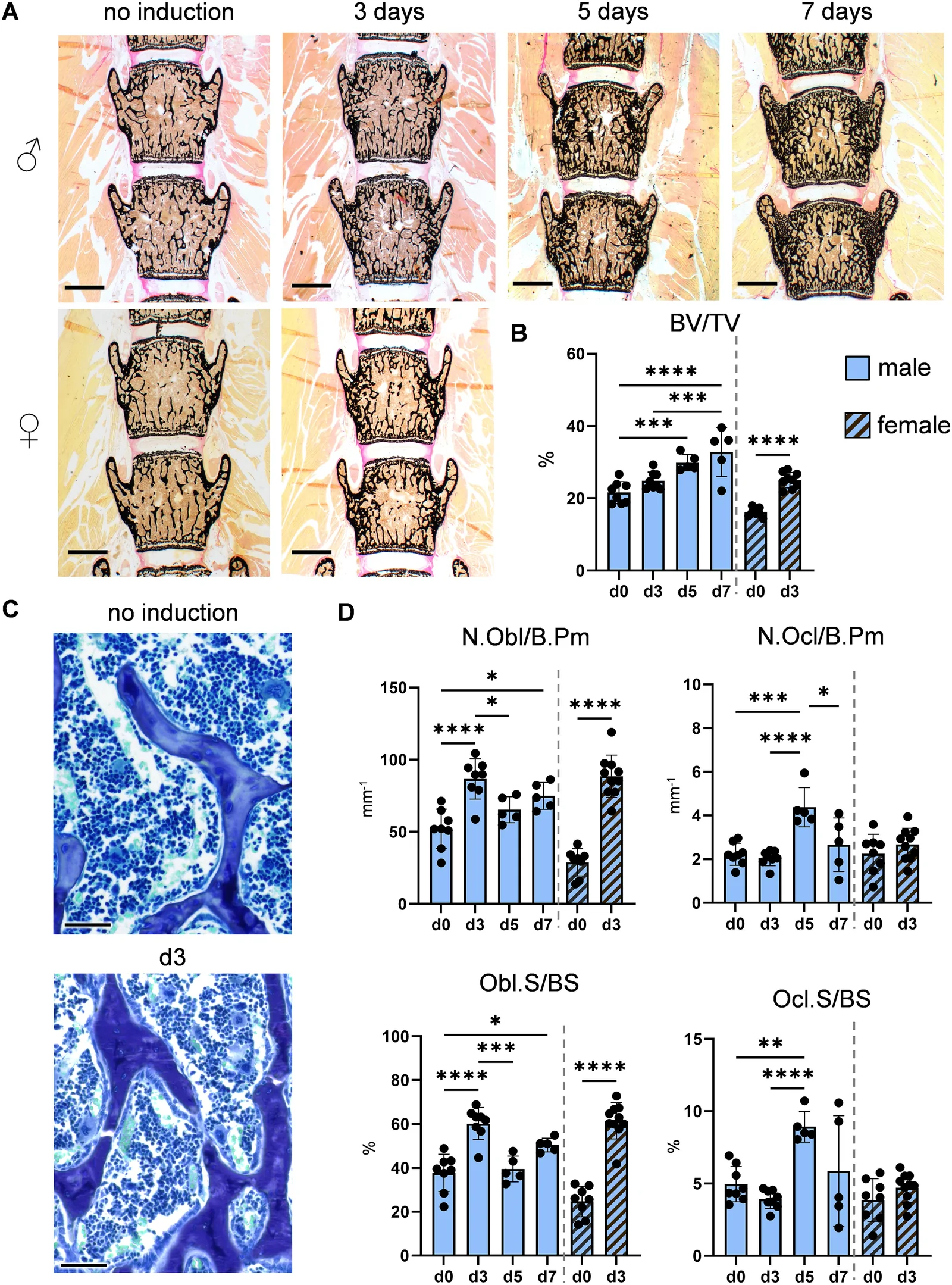
Wnt1 causes rapid bone formation driven by increased numbers of osteoblasts. (A) Representative undecalcified von Kossa/van Gieson stained sections of vertebral bodies from 6-wk-old male (♂) or female (♀) mice with no or 3, 5, or 7 d induction of the *Wnt1* transgene prior to sacrifice. Scale bar = 1 mm. (B) Histomorphometric quantification of the trabecular bone volume (BV/TV), of the vertebral bodies L3 and L4 from the mice described in the previous subpanel. Male mice d0: *n* = 8; d3: *n* = 7; d5: *n* = 5; d7: *n* = 5. Female mice: d0: *n* = 8; d3: *n* = 10. (C) Representative high magnification images of undecalcified toluidine blue stained sections of vertebral bodies from 6-wk-old male mice with no or 3 d induction of the *Wnt1* transgene prior to sacrifice. Scale bar = 50 μm. (D) Histomorphometric quantification of cellular parameters of the vertebral body L4 from 6-wk-old male or female mice with no or 3, 5, or 7 d induction of the *Wnt1* transgene prior to sacrifice. Each symbol represents an individual mouse. Data were analyzed by one way ANOVA with Tukey’s multiple comparison test. ^*^p < .05, ^**^p < .01, ^***^p < .001, ^****^p < .0001.

As we have previously demonstrated that the induction of the osteoblast-specific *Wnt1* transgene causes a significant increase of bone mass within 7 d, we have now set out to explore the timeline of this process at a higher temporal resolution. We therefore analyzed 6-wk-old male mice, in which *Wnt1* expression was either not induced or induced for 3, 5, or 7 d. To support our findings for the shortest duration of *Wnt1* induction (3 d), we also analyzed female mice. Structural histomorphometric analysis of lumbar vertebral bodies from 6-wk-old male mice revealed that, compared to non-induced controls, the overall trabecular bone mass was significantly increased after 5 and 7 d of *Wnt1* induction (Figure 1A and B). Whereas a shorter *Wnt1* induction did not yet affect trabecular bone mass in male mice, there was a significant increase in female animals already after 3 d of *Wnt1* induction.

Cellular histomorphometry showed that the number of osteoblasts, as well as the bone surface covered by osteoblasts, were strongly and significantly increased by *Wnt1* induction (Figure 1C). This influence was observed already after 3 d of *Wnt1* induction in both, male and female mice. Surprisingly, however, as assessed in male animals, this was only a temporary peak in osteoblast parameters. More specifically, there was no significant difference toward non-induced animals after 5 d, and only after 7 d the osteoblast parameters were again significantly higher than in non-induced animals (Figure 1D). Consistently, we also observed a time-dependent increase of circulating osteocalcin levels (Figure S1). With respect to osteoclasts we observed a peak in osteoclast number and surface occurring after 5 d of *Wnt1* induction, potentially indicative of a coupling effect secondary to the previous peak of osteoblast parameters (Figure 1D).

**Figure 2:**
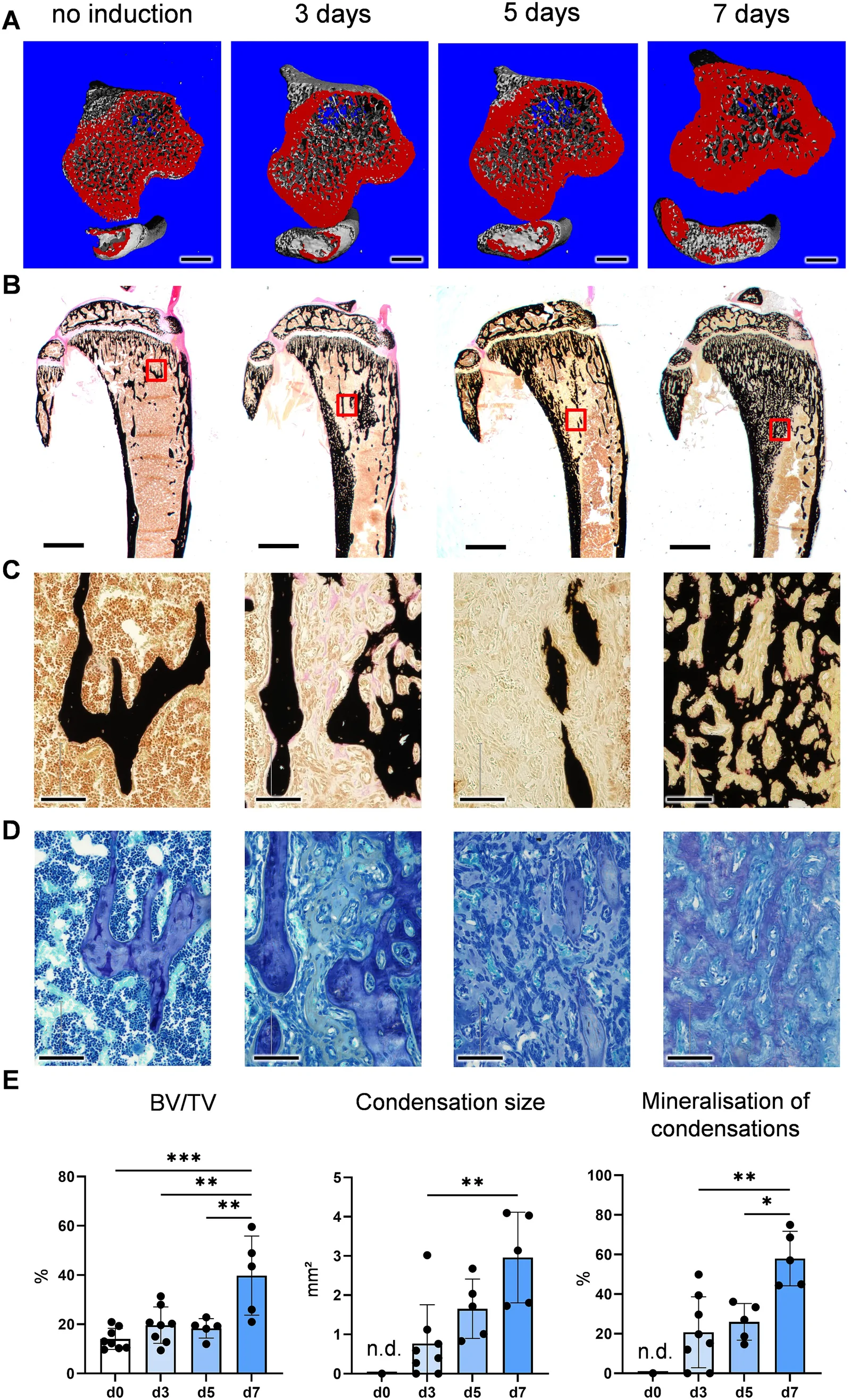
The Wnt1-induced osteoanabolic response predominantly originates from the endocortical surface. (A) Representative micro-CT images of tibiae from 6-wk-old male mice with 3, 5, 7, or no induction of the Wnt1-transgene prior to sacrifice. Axial view from the proximal toward the distal metaphysis. The 3D reconstruction is virtually cut approx. 2 mm distal of the proximal growth plate. The virtual cut plane appears red. Scale bar = 500 μm. (B) Representative undecalcified von Kossa/van Gieson stained tibia sections from the same mice. Scale bar = 1 mm. Red squares indicate the regions magnified in (C), showing von Kossa/van Gieson staining and (D), showing toluidine blue staining. Scale bar = 100 μm. (E) Quantification of the mineralized matrix in the proximal metaphysis (BV/TV), the size of dense cellular and connective tissue agglomerations/condensations, and the relative mineralization of these condensations in 6-wk-old male mice with no or 3, 5, or 7 d induction of the Wnt1-transgene. n.d., non-detectable. Each symbol represents an individual mouse. d0: *n* = 8; d3: *n* = 7; d5: *n* = 5; d7: *n* = 5. Data were analyzed by one-way ANOVA with Tukey’s multiple comparison test. ^*^p < .05, ^**^p < .01, ^***^p < .001, ^****^p < .0001.

We next investigated the influence of short-term *Wnt1* induction on long bones (tibia), where we first applied μCT scanning. In the proximal metaphysis, we observed an apparently increased bone mass with densely packed structures forming from the endocortical surface and progressing into the medullary space (Figure 2A). However, due to the unusual morphological appearance, a clear distinction between cortical and trabecular bone was unreliable, thus preventing quantitative analysis. We therefore focused on the analysis of undecalcified tibia sections, in which we confirmed the remarkable bone mass increase in male mice after 7 d of *Wnt1* induction (Figure 2B). Importantly, however, careful evaluation of the sections revealed that non-mineralized tissue agglomerations preceded this increase in mineralized matrix (Figure 2C). More specifically, areas of tissue condensation, enriched in cells morphologically resembling mesenchymal osteoprogenitors, were already detectable after 3 d of *Wnt1* induction (Figure 2D). Since there was no difference between male and female mice in this aspect, we focused our quantitative analysis on the time-dependent influence of *Wnt1* induction in the male group. Here, the standardized determination of the relative bone mass revealed an increase of mineralized trabecular bone only after 7 d of *Wnt1* induction (Figure 2E). With respect to the mesenchymal cell condensations, we identified a steady accumulation, eventually resulting in a mineralized matrix. In combination with the apparent partial trabecularization of the cortical bone that was visible by μCT scanning, it can be concluded that these structures originate from the endocortical bone surface. Taken together, our data suggested that induction of the *Wnt1* transgene causes a rapid increase in bone mass which appears to be driven by recruitment of osteoprogenitor cells.

### ST2 and MC3T3-E1 cells differentially respond to recombinant Wnt1

**Figure 3:**
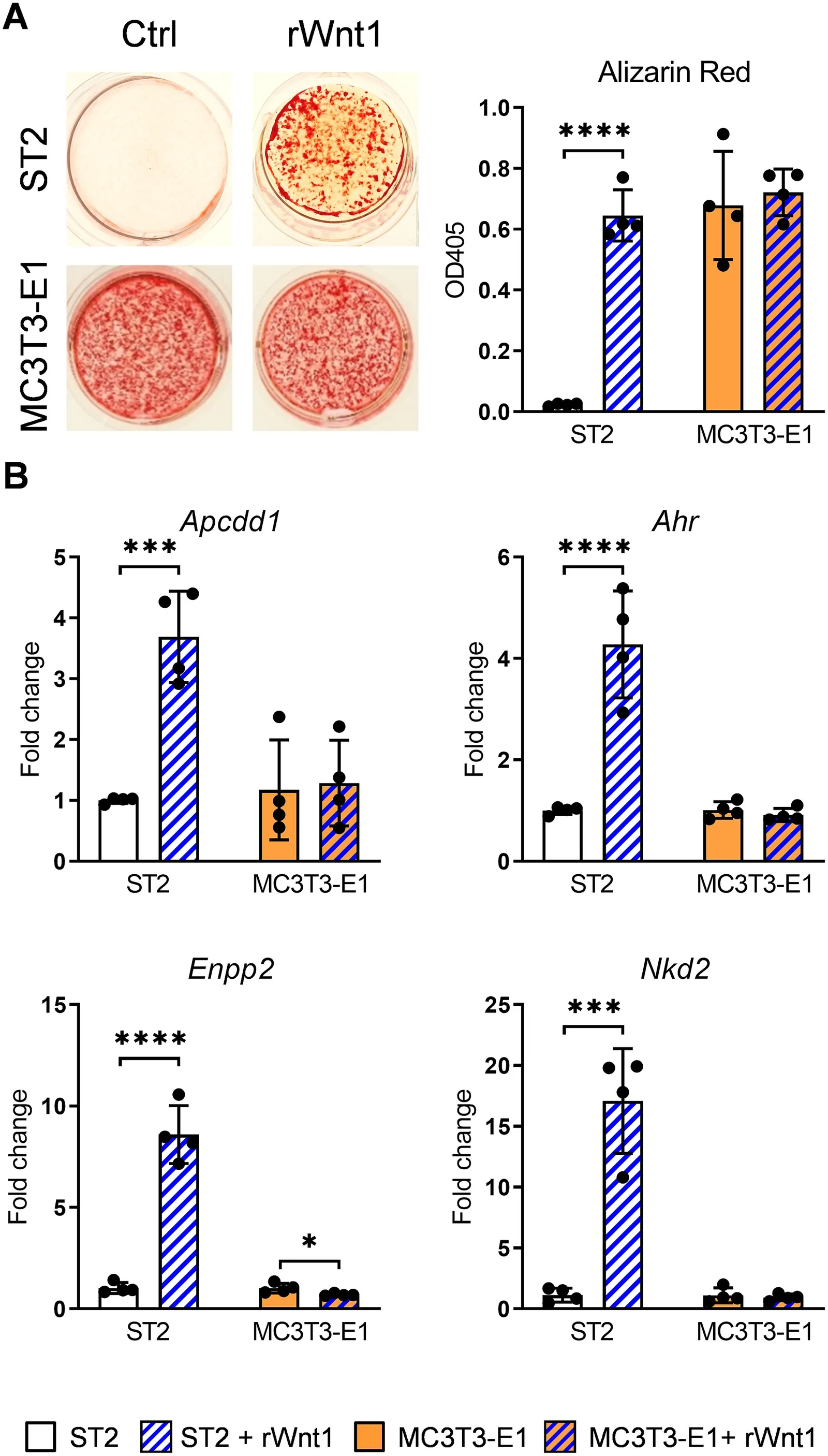
ST2 cells react to Wnt1, while MC3T3-E1 cells do not. (A) Representative images of alizarin red stained ST2 and MC3T3-E1 cell cultures after 15 d of osteogenic differentiation continuously supplemented with PBS or 100 ng recombinant Wnt1 complex (rWnt1). Photometric quantification of the alizarin red staining. (B) Gene expression analysis of *Apcdd1, Ahr, Enpp2*, and *Nkd2* by qRT-PCR in ST2 and MC3T3-E1 cell cultures after 6 h of treatment with recombinant Wnt1 (rWnt1) or PBS. Each symbol represents an individual sample. Data were analyzed by Student’s *t-*test. ^***^p < .001, ^****^p < .0001.

To confirm this hypothesis and to investigate the influence of Wnt1 on osteoprogenitor cells, we took advantage of the mesenchymal cell line ST2, which was compared to the preosteoblastic cell line MC3T3-E1. Our reasoning to focus on cell lines was that *Wnt1* induction^11^ or Wnt1 administration (Figure S2A) did not enhance osteogenesis in primary calvarial osteoblasts. Moreover, since we had previously shown that inactivation of Fzd9, the most strongly induced putative Wnt1 receptor during primary osteoblast differentiation (Figure S2B),^27^ did not impair the osteoanabolic response toward Wnt1 induction in vivo,^9^ we particularly focused on ST2 cells, since these were previously reported to respond to Wnt1 administration.^9^^,^^11^^,^^12^ We first analyzed the osteogenic potential of Wnt1 by supplementing the differentiation medium with a previously described recombinant Wnt1 complex,^9^^,^^28^ and assessed matrix mineralization after 15 d. Here, we observed that osteogenic differentiation of ST2 cells can be induced by Wnt1, whereas matrix mineralization was not enhanced by Wnt1 supplementation in MC3T3-E1 cells (Figure 3A). We further determined the expression of the established Wnt1 target genes *Apcdd1, Ahr, Enpp2*, and *Nkd2* by qRT-PCR in both cell lines. More specifically, in a previously performed transcriptome analysis after short-term Wnt1 administration to ST2 cells, these 4 genes were found to be most strongly induced.^9^ In the present study, we observed that all 4 target genes were significantly induced in ST2 cells after 6 h treatment with recombinant Wnt1, while this effect was completely absent in MC3T3-E1 cells (Figure 3B). In contrast, treatment with recombinant Wnt3a for 6 h resulted in similar inductions of these target genes in both cell lines, demonstrating that MC3T3-E1 cells are in principle capable of responding to canonical Wnt signals (Figure S3). Overall, these findings were not only consistent with our in vivo observations, they also enabled a screening for a putative Wnt1 receptor with osteoanabolic function, by comparing gene expression in responsive ST2 and non-responsive MC3T3-E1 cells.

**Figure 4:**
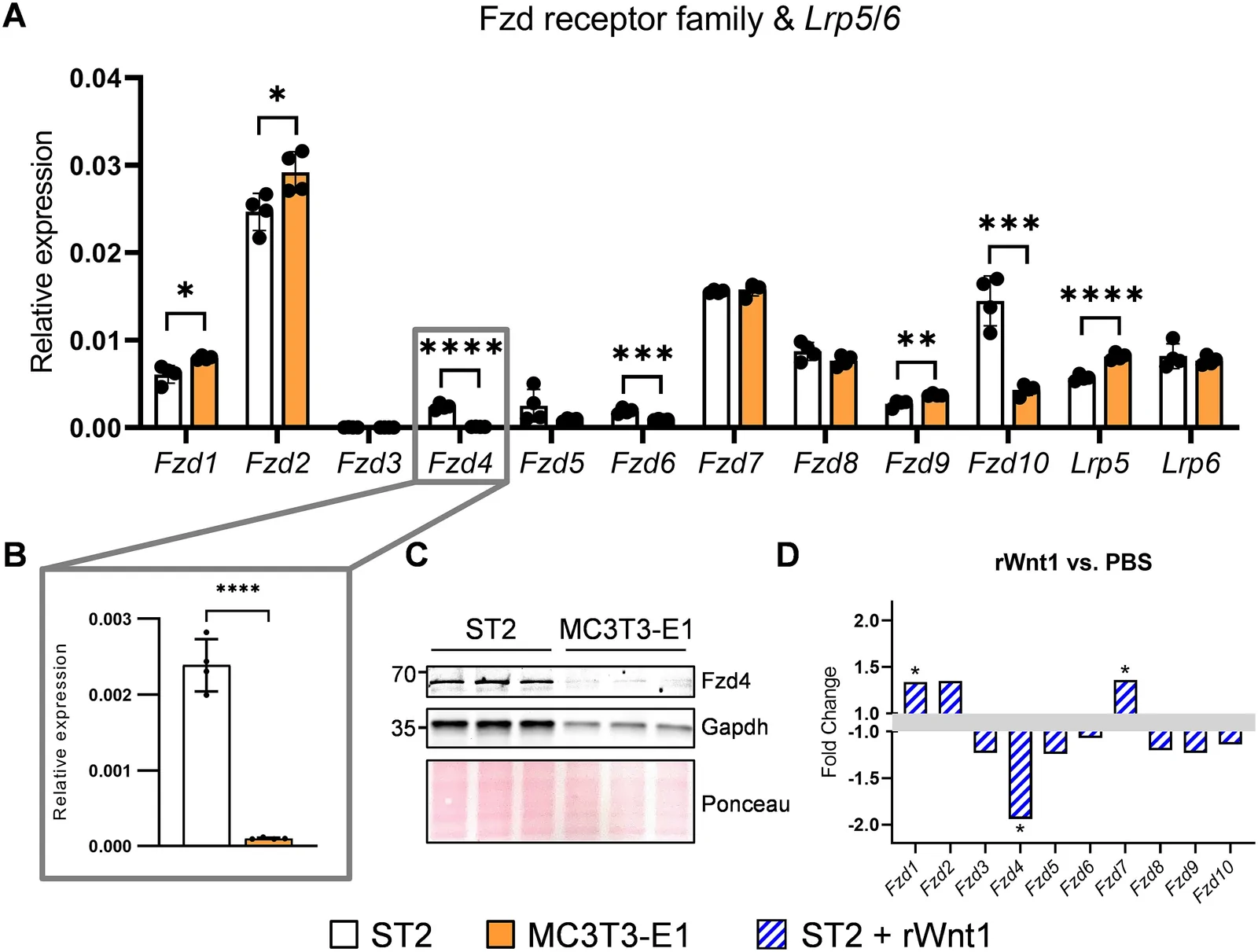
*Fzd4, Fzd6*, and *Fzd10* display lower expression in MC3T3-E1 cells than in ST2 cells. (A) Expression analysis of all *Fzd* genes and *Lrp5* and *Lrp6* in ST2 and MC3T3-E1 cells by qRT-PCR. Each symbol represents an individual sample. Data were analyzed by Student’s *t-*test. ^*^p < .05, ^**^p < .01, ^***^p < .001, ^****^p < .0001. (B) Magnified graph of the expression pattern of *Fzd4* from the previous subpanel. (C) Western blot analysis of Fzd4 protein content in ST2 and MC3T3-E1 cells with Gapdh and Ponceau S staining as loading controls. (D) Relative expression of all *Fzd* genes in ST2 cells stimulated for 6 h with recombinant Wnt1 compared to PBS-treated controls as determined by microarray whole transcriptome analysis with 3 samples per condition. Data were analyzed by one-way ANOVA, ^*^p < .05.

As a first step, we determined the expression of all known FZD receptors as well as the co-receptors *Lrp5* and *Lrp6* in both cell lines. We found that 3 *Fzd* genes, namely *Fzd4, Fzd6*, and *Fzd10*, displayed significantly lower expression in MC3T3-E1 cells (Figure 4A). The strongest difference was observed for Fzd4, which was also confirmed at the protein level. More specifically, while Fzd4 could be detected in ST2 cells by Western blotting, this receptor was nearly absent in MC3T3-E1 cells (Figure 4C). To explore potential feedback mechanisms and gain more robust insights into the transcriptional response, we performed a microarray-based genome wide expression analysis with biological triplicates for ST2 cells treated for 6 h with recombinant Wnt1 or PBS as controls. Here, we observed a significant differential expression of more than 150 genes, and we were able to confirm the induction of our selected Wnt1 target genes *Apcdd1, Ahr, Enpp2*, and *Nkd2* (Figure S4A-C)*.* Interestingly, some other established Wnt signaling target genes were not induced by Wnt1 in ST2 cells, and for *Axin2* expression, this was also the case for Wnt3a. In MC3T3-E1 cells, however, *Axin2* expression was moderately induced by Wnt1 and strongly induced by Wnt3a, which essentially demonstrates that such transcriptional regulations strongly depend on both, the cellular context and the specific Wnt ligand added (Figure S4D)*.* More importantly, among all *Fzd* genes, only *Fzd4* was significantly repressed in response to recombinant Wnt1 treatment (Figure 4D), potentially indicating a negative feedback mechanism and further supporting the hypothesis that Fzd4 could serve as a physiologically relevant receptor for Wnt1.

### Fzd4, Lrp5, and Lrp6 are required for the molecular response of ST2 cells toward Wnt1

**Figure 5:**
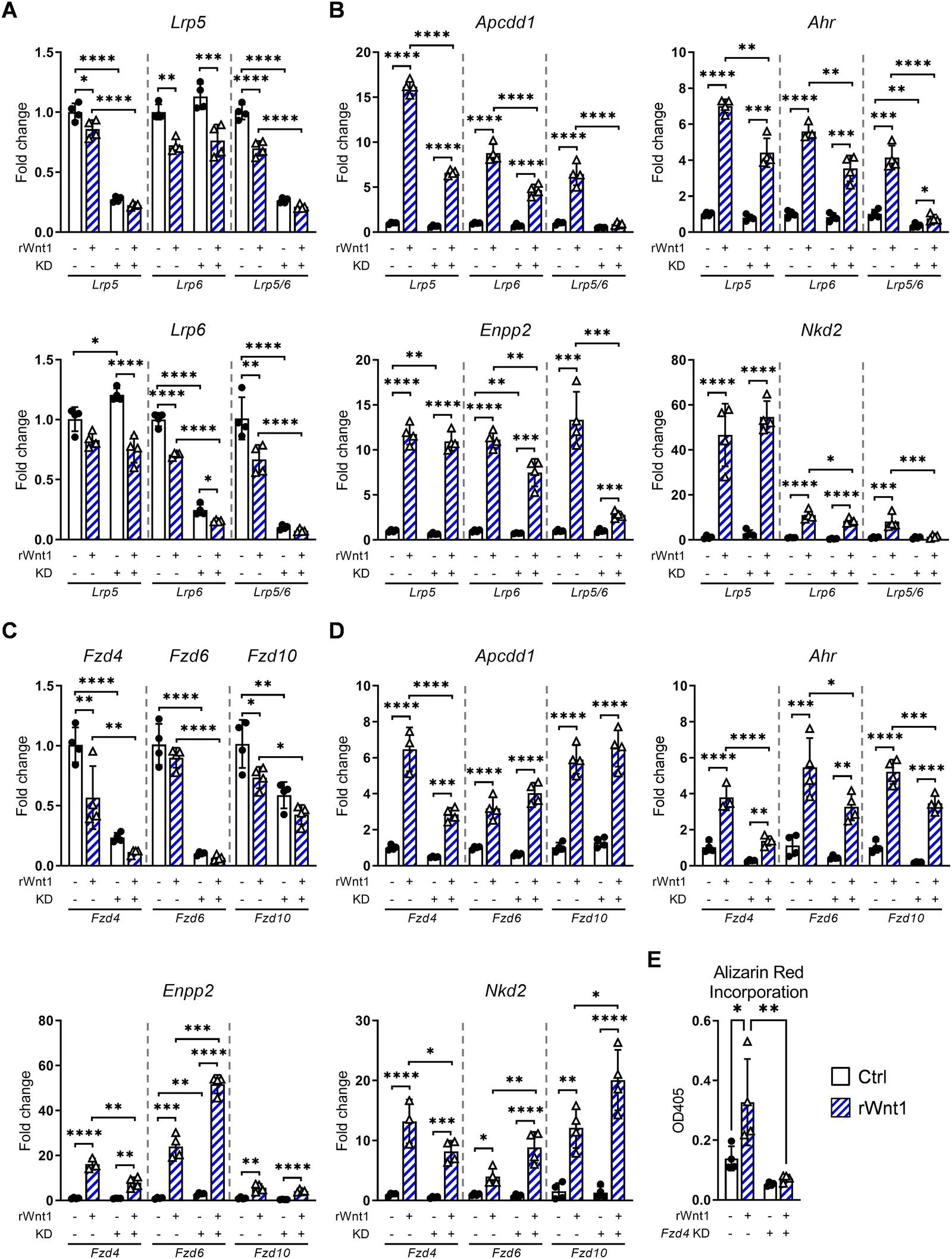
Silencing of *Lrp5* and *Lrp6* or *Fzd4* impairs the transcriptional response toward Wnt1 in ST2 cells. (A) Expression analysis of *Lrp5* and *Lrp6* by qRT-PCR in ST2 cells treated with PBS (Ctrl, −) or recombinant Wnt1 (rWnt1, +) for 6 h after siRNA mediated silencing (knockdown, KD, +) of *Lrp5, Lrp6* or both *Lrp5* and *Lrp6* 48 h prior to treatment. (B) Expression analysis of *Apcdd1, Ahr, Enpp2*, and *Nkd2* by qRT-PCR in the same samples. (C) qRT-PCR based expression analysis of *Fzd4, Fzd6*, and *Fzd10* in ST2 cells treated with PBS (Ctrl, −) or recombinant Wnt1 (rWnt1, +) for 6 h after siRNA mediated silencing (knockdown, KD, +) of *Fzd4, Fzd6*, and *Fzd10*, respectively, 48 h prior to treatment. (D) Expression analysis of *Apcdd1, Ahr, Enpp2*, and *Nkd2* by qRT-PCR in the same samples as in subpanel C. (E) Photometric quantification of alizarin red stained ST2 cell cultures with siRNA mediated silencing of *Fzd4* expression (knockdown, KD, +) after 10 d of osteogenic differentiation continuously supplemented with PBS or 100 ng recombinant Wnt1 complex (rWnt1, +). Each symbol represents an individual sample. Data were analyzed by 2-way ANOVA with Sidak’s multiple comparison test. ^*^p < .05, ^**^p < .01, ^***^p < .001, ^****^p < .0001.

To obtain further insights into a possible receptor complex mediating the osteoanabolic influence of Wnt1, we next performed siRNA-mediated knockdown experiments. Here, we first used siRNAs targeting the co-receptors Lrp5 and Lrp6, either alone or in combination. Quantitative RT-PCR analysis showed that the siRNA-mediated knockdown of Lrp5 and Lrp6, individually and in combination, was effective and resulted in a strong and significant reduction of expression (Figure 5A). While individual silencing of either Lrp5 or Lrp6 resulted in a partially diminished, but not fully abrogated induction of the target genes *Apcdd1, Ahr, Enpp2*, and *Nkd2* upon Wnt1 treatment, the combined knockdown of both, *Lrp5* and *Lrp6*, strongly blocked the expression of all four genes in Wnt1-treated ST2 cells (Figure 5B). These findings demonstrate functional redundancy of Lrp5 and Lrp6 in the response of ST2 cells toward Wnt1, which can also explain our previous observation that Lrp5 deficiency did not impair the osteoanabolic influence toward transgenic *Wnt1* induction in vivo*.*^11^

To evaluate, which Fzd receptor is required to convey the signal induced by Wnt1 in ST2 cells, we next performed siRNA-mediated knockdown of *Fzd4, Fzd6*, and *Fzd10*, the 3 *Fzd* genes with significantly lower expression in MC3T3-E1 cells. We found that the siRNA-mediated knockdown was highly effective for *Fzd4* and *Fzd6*, while the expression of *Fzd10* was reduced to a lesser, although still significant extent (Figure 5C). With regard to the expression of *Apcdd1* after 6 h of recombinant Wnt1 treatment, only cells subjected to the *Fzd4*-specific siRNA displayed a diminished, although not fully absent response (Figure 5D). When considering the expression of the target gene *Ahr*, the knockdown of all 3 candidate receptors *Fzd4, Fzd6*, and *Fzd10* caused a significant reduction in *Ahr* induction upon Wnt1 treatment, yet the knockdown of *Fzd4* had again the largest effect (Figure 5D). Moreover, only the knockdown of *Fzd4* significantly reduced the induction of *Nkd2* and *Enpp2*, while it was unaffected or even enhanced by silencing of *Fzd6* or *Fzd10* (Figure 5D). Additionally, silencing of *Fzd4* removed the ability of ST2 cells to form mineralized matrix in the presence of osteogenic medium and recombinant Wnt1 (Figure 5E), providing a functional line of evidence. Taken together, these results indicated that Fzd4 is required for a full molecular and functional response of ST2 cells toward Wnt1. In this regard, it is also interesting to state that *Fzd4* expression was strongly reduced in ST2 cells by the combined knockdown of *Lrp5* and *Lrp6* (Figure S5)*.* Based on our experimental evidence, but also supported by the previously reported osteopenia in mice with an osteoblast-specific Fzd4 inactivation,^29^ we hypothesized that FZD4 is a physiologically relevant Wnt1 receptor with osteoanabolic function.

### Low bone mass in mice with heterozygous *Fzd4* inactivation

**Figure 6:**
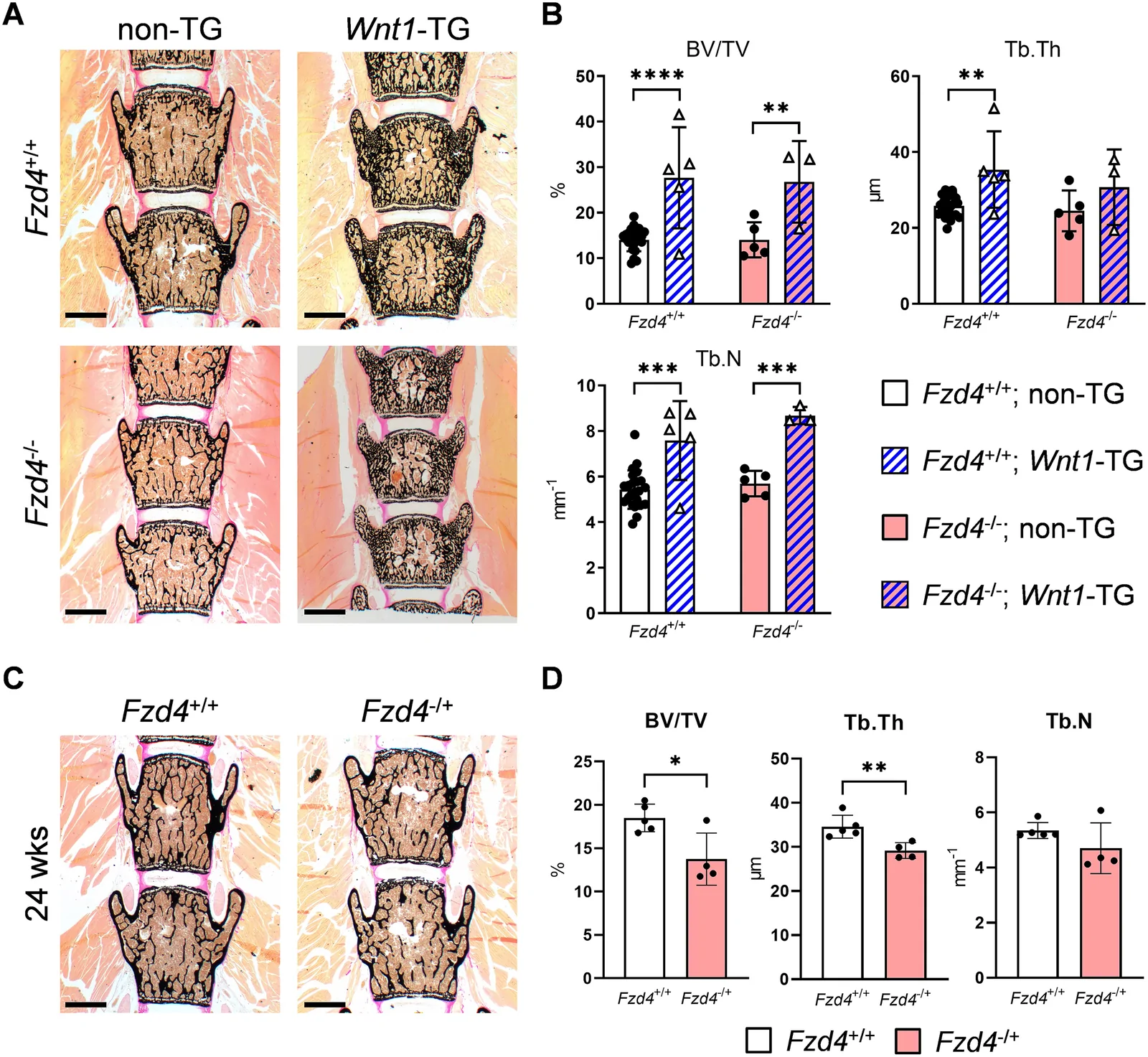
FZD4 deficiency results in osteopenia, but does not impair the osteoanabolic response to *Wnt1* induction. (A) Representative undecalcified von Kossa/van Gieson stained spine sections from 5-wk-old mice with the indicated *Fzd4* and *Wnt1*-TG genotypes after 7 d of induction of the Wnt1 transgene. (B) Histomorphometric quantification of the trabecular bone volume (BV/TV), trabecular thickness (Tb.Th), and trabecular number (Tb.N) of the vertebral bodies L3 and L4 from 5-wk-old *Fzd4*^+/+^ and *Fzd4*^−/−^ mice with and without the *Wnt1* transgene after 7 d of transgene induction where applicable. Each symbol represents an individual mouse. *Fzd4*^+/+^; non-TG: *n* = 23, *Fzd4*^+/+^; *Wnt1*-TG: *n* = 5, *Fzd4*^−/−^; non-TG: *n* = 5; *Fzd4*^−/−^; *Wnt1*-TG: *n* = 3. Data were analyzed by two-way ANOVA with Sidak’s multiple comparison test. ^**^p < .01, ^***^p < .001, ^****^p < .0001. (C) Representative undecalcified von Kossa/van Gieson stained spine sections from 24-wk-old *Fzd4* WT (+/+) or heterozygous (+/−) female mice. (D) Histomorphometric quantification of structural parameters in the vertebral bodies L3 and L4 from 24-wk-old female *Fzd4*^+/+^ and *Fzd4*^−/+^ mice. Each symbol represents an individual mouse. *Fzd4*^+/+^: *n* = 5; *Fzd4*^−/+^: *n* = 4. Data were analyzed by Student’s *t-*test. ^*^p < .05, ^**^p < .01.

In a first attempt to address this possibility, we introduced the inducible *Wnt1* transgene into a *Fzd4*-deficient mouse line. Due to the limited life expectancy of *Fzd4*-deficient animals, we focused our investigation on 5-wk-old mice, in which *Wnt1* expression was induced for 7 d. Although we only obtained a limited number of animals, our histomorphometric analysis of vertebral body sections clearly showed that *Fzd4* inactivation does not impair the osteoanabolic effect of *Wnt1* induction in vivo (Figure 6A and B). Similar findings were obtained by histomorphometric analysis of undecalcified tibia sections (Figure S6). In conclusion, although *Fzd4*-deficient mice are smaller and display premature death,^30^ their response toward the osteoanabolic influence of Wnt1 is not impaired, thereby questioning the physiological relevance of Fzd4 as a putative Wnt1 receptor. Importantly, however, since we have previously observed the same negative result, when we analyzed *Lrp5*-deficient *Wnt1*-transgenic mice,^11^ we speculate that this finding might be explained by compensatory mechanisms, possibly involving FZD6 and/or FZD10. Especially in the few surviving *Fzd4*-deficient *Wnt1*-transgenic animals, there might be a confounding survivor bias.

Since osteoblast-specific inactivation of *Fzd4* has already been reported to result in impaired trabecular bone formation,^29^ we finally addressed another physiologically relevant question, namely if *Fzd4* haploinsufficiency would cause a low bone mass phenotype. We therefore analyzed undecalcified spine sections from 24-wk-old male *Fzd4*-heterozygous mice and their WT littermates. Here, we identified a moderate, yet significant decrease of trabecular bone mass in *Fzd4*-heterozygous animals, similar to what has been described for Lrp5-deficient mice (Figure 6C and D).^31^^,^^32^

## Discussion

Wnt1 has emerged as a key regulator of bone mass as exemplified by skeletal disorders that are caused by pathogenic variants of *WNT1* (early-onset osteoporosis and osteogenesis imperfecta type XV) and supported by evidence from loss-of-function and gain-of-function mouse models.^6–15^ One important observation in this context was that Wnt1 induced a strong osteoanabolic response in an inducible osteoblast-specific transgenic mouse model.^11^ A molecular understanding of the Wnt1 action, and particularly, the identification of the relevant receptor complex and target cells, could be an important step in terms of developing of novel osteoanabolic treatment options.

In the present study, we could show that the induction of the osteoblast-specific *Wnt1* transgene causes a significant increase of bone mass within 3 d in female and within 5 d in male mice, with a strong but transient increase in osteoblasts already discernible 3 d after induction, followed by a transient increase in osteoclast parameters at day 5. The transient nature of the observed increase in parameters favors the hypothesis that Wnt1 initially stimulates the differentiation or activation of an already existing pool of precursor cells, which is depleted after the initial burst of differentiation. As we have previously demonstrated that also after 3 wk of *Wnt1*-induction, a significant increase of osteoblast parameters was observable, it appears likely that in the context of continued Wnt1 stimulation, the bone metabolism adapts to these altered demands by expanding the stem cell pool itself. Although we analyzed female mice only after 3 d of *Wnt1* induction, it is also important to state that their immediate response in vertebral bodies was even more pronounced than observed in male animals. In our opinion, this difference might be explained by the relatively lower bone mass in non-induced female animals. Nonetheless, the data obtained in the current study further highlight the gender-independent osteoanabolic potential of Wnt1 and indicate that the first skeletal response to elevated Wnt1 signaling is a rapid recruitment and activation of osteoprogenitor cells that differentiate into active osteoblasts. Alternatively, the observed increase in osteoblasts may also originate from the activation and proliferation of bone lining cells.

In order to fully exploit the therapeutic potential of Wnt1, it is of paramount importance to identify the relevant receptor complex. Importantly, we have identified two cell lines with the potential to provide further insights, since one of them responds to recombinant Wnt1 (ST2 mesenchymal cells), whereas the other does not (MC3T3-E1 osteoblasts). Indeed, comparing the expression all Fzd receptors in these 2 cell lines revealed that only *Fzd4, Fzd6*, and *Fzd10* are expressed at a higher level in the responding ST2 cells than in MC3T3-E1 cells. Our knockdown experiments have further shown that reduction of *Fzd4, Fzd6*, and *Fzd10* expression can all cause a decreased response to Wnt1 stimulation in ST2 cells. However, regarding the degree of reduction and consistency throughout all analyzed target genes, FZD4 appeared to be the most relevant candidate receptor. Additional observations, such as an apparent negative feedback loop observed in our transcriptomic analysis and abrogated ability to form mineralized matrix in the presence of recombinant Wnt1 after silencing of *Fzd4*, further emphasized the potential role of FZD4 as the predominant receptor for Wnt1. Additionally, in silico docking simulations of Wnt1 with several members of the Fzd family indicated that FZD4 is among the most likely interaction partners of Wnt1.^33^ One limitation of our investigation is the use of immortalized cell lines. Indeed, future experiments, exploring whether Wnt1 elicits a similar transcriptional response in primary bone marrow mesenchymal stromal cells as in ST2 cells, could support the physiological relevance and transferability of our data. A first indication toward the in vivo relevance of our findings is the observation that all four Wnt1-target genes, *Apcdd1, Ahr, Enpp2*, and *Nkd2*, that were used as a primary readout in this study, were also significantly induced in fracture calli of Wnt1-transgenic mice.^16^

However, by combining the *Wnt1*-transgene with Fzd4 deficiency, we were not able to support the direct interaction of Wnt1 and FZD4 in vivo. One major limitation that needs to be considered in this context is however a potential survivor bias due to the reduced viability of *Fzd4*-deficient mice. Indeed, it is possible that the few animals that survived until analysis activated alternative mechanisms to compensate for the loss of FZD4. On the other hand, we cannot rule out that other receptors are more relevant in mediating the osteoanabolic influence of Wnt1 in vivo. In this context, it is however important to state that we have previously observed that Lrp5 deficiency does not impair the osteoanabolic response to transgenic *Wnt1* induction either.^11^ Our initial hypothesis was that Lrp6, rather than Lrp5, would serve as a physiologically relevant co-receptor for the osteoanabolic function of Wnt1. Based on our siRNA knockdown experiments presented here, it is however more likely that both co-receptors can mediate this function and that Lrp6 can fully compensate for the loss of Lrp5 in the context of Wnt1-induced bone formation. This conclusion is also supported by findings in mouse deficiency models, indicating that both co-receptors have specific and overlapping functions in bone formation.^34^^,^^35^

With respect to a putative receptor complex with osteoanabolic function, it is also important to refer to human genetic evidence. In fact, *FZD*4 has not only been associated with bone mass in GWAS studies, but it was also identified as a disease gene.^18^^,^^19^^,^^36–39^ More specifically, heterozygous pathogenic variants of *FZD4* have been found to cause familial exudative vitreoretinopathy (FEVR), and this disease can also be caused by heterozygous pathogenic variants of *LRP5*.^18^^,^^19^ However, unlike it is routinely performed for FEVR patients with *LRP5* variants, affected patients with *FZD4* variants have not been systematically screened for skeletal pathologies so far. In our opinion, since a Norrin-dependent interaction of Lrp5 and FZD4 has already been established in the context of retinal angiogenesis,^20^ it will be important to investigate if heterozygous loss-of-function variants of *FZD4* result in early-onset osteoporosis. Addressing this question is also relevant, since a FZD4/Lrp5 agonist (SZN-413) has already been developed for the treatment of diabetic retinopathy, which was shown to be effective in preclinical trials using mice and rabbits.^40^ Therefore, we are convinced that our findings are an important first step with respect to optimizing the still limited osteoanabolic treatment options for patients with low bone mass disorders.

## Supplementary Material

Supplementary_material_ziag153

## Data Availability

All relevant data are presented in the manuscript; raw data are available upon reasonable request from the corresponding author. Microarray data is available in the GEO repository (GSE311746).
