# Supplementary material for: Identification of candidate receptors mediating the osteoanabolic influence of Wnt1
Source: JBMR Plus. 2026 Sep 9;10(10):ziag153. doi: 10.1093/jbmrpl/ziag153 (PMC13592163; doi:10.1093/jbmrpl/ziag153)
Supplement: Supplementary file 1 [file supplementary_material_ziag153.zip › Supplementary Figure.docx]

**Supplementary Figure 1**

Serum Osteocalcin levels in 6-week-old male mice with 3, 5, 7 or no induction of the Wnt1-transgene prior to sacrifice as determined by an enzyme-linked immunosorbent assay. Each symbol represents an individual mouse. Data were analyzed by one way ANOVA with Tukey’s multiple comparison test. *p<0.05

**Supplementary Figure 2**

Wnt1 and Fzd receptors in primary osteoblasts A) Representative images of alizarin red stained primary murine wildtype calvarial osteoblast cultures after 10 days of osteogenic differentiation continuously supplemented with PBS or 100 ng recombinant Wnt1 complex (rWnt1). Photometric quantification of the alizarin red staining. B) Gene expression analysis by qRT-PCR of all Fzd receptors in primary murine calvarial osteoblasts (wildtype) after 0, 5, 10, 15 and 20 days of osteogenic differentiation. Data are shown relative to *Gapdh* expression. Each symbol represents an individual sample.

**Supplementary Figure 3**

ST2 and MC3T3-E1 cells react to Wnt3a. Gene expression analysis of *Apcdd1,* *Ahr, Enpp2 and Nkd2* by qRT-PCR in ST2 and MC3T3-E1 cell cultures after 6 h of treatment with recombinant Wnt3a (rWnt3a) or PBS. Each symbol represents an individual sample. n.d. = non-detectable. Data were analyzed by Student’s t-test. ***p<0.001, ****p<0.0001.

**Supplementary Figure 4**

Microarray analysis of ST2 cells treated for 6h with recombinant Wnt1 compared to PBS treated cells. 3 biological replicates per condition. A) Number of significantly differentially expressed genes. B) Volcano plot fold change vs. one way ANOVA p-value of the microarray based gene expression analysis. Green indicates significantly repressed genes with a fold change <-2.0, red indicates significantly induced genes with a fold change >2.0. Arrows and labels point toward the four genes used as Wnt1 stimulation readouts in this study. Every dot represents one assessed gene. C) Heat map of the expression of the four selected Wnt1 target genes *Apcdd1,* *Ahr, Enpp2* and *Nkd2* as well as the established canonical Wnt target genes *Axin2*, *Tcf7* and *Lef1*. *Apcdd1* and *Ahr* were the two most induced genes in the comparison. D) Gene expression analysis of *Axin2* by qRT-PCR in ST2 and MC3T3-E1 cell cultures after 6 h of treatment with recombinant Wnt1 (rWnt1) or PBS. E) Gene expression analysis of *Axin2* by qRT-PCR in ST2 and MC3T3-E1 cell cultures after 6 h of treatment with recombinant Wnt3a (rWnt3a) or PBS. Each symbol represents an individual sample. Data were analyzed by Student’s t-test. *p<0.05, ***p<0.001.

**Supplementary Figure 5**

Fzd receptor response to *Lrp5/6* silencing. Gene expression analysis of all Fzd receptors in ST2 cells 48 hours after siRNA mediated silencing (knockdown, KD) of both *Lrp5*and *Lrp6*. Each symbol represents an individual sample. Data were analyzed by Student’s t-test. *p<0.05, **p<0.01, ***p<0.001, ****p<0.0001.

**Supplementary Figure 6**

FZD4 deficiency reduces body length but does not impair the osteoanabolic response to *Wnt1* induction in tibiae. A) Representative undecalcified von Kossa/van Gieson stained tibia sections from 5-week-old mice with the indicated *Fzd4* and *Wnt1*-TG genotypes after 7 days of induction of the Wnt1 transgene. B) Histomorphometric quantification of the trabecular bone volume (BV/TV), trabecular thickness (Tb.Th) and trabecular number (Tb.N) in the proximal metaphysis of tibiae from 5-week-old *Fzd4*^+/+^ and *Fzd4*^-/-^ mice with and without the *Wnt1* transgene after 7 days of transgene induction where applicable. C) Body length (torso) of the same mice. Each symbol represents an individual mouse. *Fzd4*^+/+^; non-TG: n=23, *Fzd4*^+/+^; *Wnt1*-TG: n=5, *Fzd4*^-/-^; non-TG: n=5; *Fzd4*^-/-^; *Wnt1*-TG: n=3. Data were analyzed by two way ANOVA with Sidak’s multiple comparison test. **p<0.01, ***p<0.001, ****p<0.0001
